# Editorial: World No-Tobacco: effects of tobacco and nicotine on the brain

**DOI:** 10.3389/fphar.2025.1610178

**Published:** 2025-05-06

**Authors:** Isis Nem de Oliveira Souza, Graciela N. Balerio, Vijayapandi Pandy, Massimo Grilli, Gilda Angela Neves

**Affiliations:** ^1^ Laboratório de Farmacologia Molecular, Instituto de Ciências Biomédicas, Universidade Federal do Rio de Janeiro, Rio de Janeiro, Brazil; ^2^ Instituto de Investigaciones Farmacológicas (ININFA-CONICET) and Cátedra de Farmacología, Facultad de Farmacia y Bioquímica, Universidad de Buenos Aires, Buenos Aires, Argentina; ^3^ Department of Pharmaceutical Sciences, School of Health Sciences and Technology, Dr. Vishwanath Karad, MIT World Peace University, Pune, India; ^4^ Department of Pharmacy, University of Genoa, Genoa, Italy

**Keywords:** tobacco and tobacco product, nicotine, vape, addiction, smoke cessation, adolescence, sex difference

Recent evidence places the earliest human tobacco use around 12,300 years ago (Duke et al., 2021). Among plants with psychoactive compounds, tobacco, and by default nicotine, is arguably the most entrenched in human history, affecting several aspects such as health, culture, and social relations (Castaldelli-Maia et al., 2016). Tobacco smoking remains the world’s leading preventable cause of death, responsible for approximately 8 million deaths per year (Reitsma et al., 2021). On World No-Tobacco Day, we are advancing our understanding of tobacco and nicotine use and its impact on human health from various perspectives, with the potential to improve public health.

Significant gaps remain in our understanding of sex differences in the development of tobacco and nicotine addiction and relapse (Kcomt et al., 2022; Davis et al., 2023). Chellian et al. investigated the operant response to nicotine and the development of addiction in adult rats of both sexes. They also evaluated whether a period of enforced abstinence affected nicotine-seeking behavior. The results showed that nicotine intake was higher in females than in males when they were given prolonged daily access to the drug, highlighting the need for more individualized approaches to tobacco cessation.

Indeed, tobacco withdrawal effects are severe enough to halt cessation efforts and require specialized help. With a success rate of around 7% (Méndez et al., 2022), understanding the mechanisms nicotine affects the reward system is fundamental to developing better strategies. Using fMRI, (Conti et al.) showed that midbrain reward-related responses are blunted in a British cohort of habitual smokers. Interestingly, they also found more pronounced abnormalities in short-term young smokers, highlighting the importance of understanding nicotine effects at different points of the life span and its long-term compensatory mechanisms for the development of comprehensive and effective smoking cessation strategies.

Recently, the most heated debate on nicotine use concerns new delivery systems. While the media freely depicts e-cigarette or vaping use by adolescents and young adults, emerging evidence suggests that their use during critical periods is associated with long-term consequences (Yuan et al., 2015). Happer et al. examined the associations between recent nicotine and tobacco product (NTP) use, primarily e-cigarettes, and bilateral hippocampal volume estimates in a sample of adolescents and young adults. Results showed that greater NTP use predicted larger hippocampal volumes but relatively lower memory scores than non-users. These findings suggest that early NTP exposure may alter typical brain-behavior relationships underlying learning and memory. Interestingly, when these findings were examined in the context of cannabis co-use, no interaction between NTP and cannabis was found. However, other studies have shown how one drug exposure can influence the response to another (Laviolette, 2021; Gonçalves et al., 2023). In this context, (Carreño et al.) investigated sex- and genotype-dependent effects of nicotine-induced methamphetamine self-administration in adolescent rats. They focused on a single-nucleotide polymorphism of the α6 nAChR subunit gene, which is well associated with higher cigarette smoking, adolescent drug experimentation, nicotine dependence, and unsuccessful quit attempts (Carreño et al., 2024). Their findings suggest functional changes in α6 nAChRs in brain regions associated with reward influenced by the CHRNA6 genotype, sex, and drug treatment. These findings provide new insights for future prevention and intervention strategies for nicotine addiction.

In addition to e-cigarettes, other unique delivery systems include nicotine pouches, which promise to be less harmful to lung health and aid in smoking cessation (Pluym et al., 2024). Mallock-Ohnesorg et al. evaluated the acute effects of different brands and doses of nicotine pouches in a German cohort of cigarette smokers. Although all pouches successfully reduced cigarette cravings, they were associated with much higher and faster nicotine intake and changes in cardiovascular parameters. These findings emphasize the urgent need for better regulation of new nicotine-releasing products to ensure their safety and effectiveness for tobacco cessation.

Returning to the topic of early life tobacco exposure, Proud et al. evaluated prenatal nicotine exposure using an *in vitro* approach. Their research confirms that nicotine exposure has not only acute but also long-term outcomes on neurogenesis and molecular markers of neural identity, mood disorders, and excitatory/inhibitory balance. Their study demonstrates how sophisticated *in vitro* approaches can contribute to neurodevelopmental research on nicotine exposure and how this exposure can be detrimental, even if not direct. Furthermore, non-smokers can still be affected by tobacco exposure through second-hand smoke (SHS), also known as passive or environmental tobacco smoke exposure.

SHS increases the risk of nine health outcomes, including ischemic heart disease, stroke, diabetes, and lung cancer. Although smoking rates have gradually declined over the past 50 years, ∼37% of the world’s population is still exposed to smoke emitted from the combustion of tobacco end-products or exhaled by smokers, with higher rates of exposure reported in women and children compared with men (Flor et al., 2024). In their review, Kisby and Raber address the Research Topic of tobacco exposure from the perspective of pathological risk, including that induced by SHS.

Alongside direct deleterious effects, nicotine use is implicated in many co-morbidities, although causal or consequential roles are rarely described (CDC-OSH: National Center for Chronic Disease Prevention and Health Promotion Office on Smoking and Health, 2014). People living with HIV are predisposed to an increased risk of developing inflammatory disorders such as HIV-associated neurocognitive disorder (HAND). Moreover, tobacco use has been observed to exacerbate further the risk of neurocognitive symptoms resulting from HIV-associated neuroinflammation (Chang et al., 2020). Indeed, there is a well-established body of literature linking HIV-1 to NLR Family Pyrin Domain Containing 3 (NLRP3) inflammasome signaling in both the periphery and the CNS. Keane and Swartz review tobacco and nicotine effects on HAND neurobiology, including effects on cognition, inflammation, viral latency, and blood-brain barrier integrity. The authors propose the NLRP3 inflammasome as a potential common pathway through which HIV-1 and nicotine may promote neuroinflammation in HIV patients.

Finally, while nicotine is predominantly recognized as the addictive component of tobacco and is linked to various smoking-related diseases, it also possesses cognitive-enhancing and anti-inflammatory properties, suggesting therapeutic potential for several conditions (Valentine and Sofuoglu, 2018; Zhang et al., 2022). The review by Cao et al. explores this dual nature of nicotine, providing a concise overview of nicotine´s physiochemical properties and pharmacology, including insights into its receptors. The discussion includes its toxic effects, which are categorized into five groups: cancer, cardiovascular, respiratory, reproductive, and others. Potential drug development applications are divided into nervous and immune interventions. All these contributions rendered a comprehensive Research Topic, and we firmly believe the readers will find this a unique and valuable reference for the state of the art in the field.
